# Switch It Off! Carbon, Financial and Health Service Impacts of Switching Off a Computed Tomography Scanner: A Quality Improvement Study

**DOI:** 10.1002/jmrs.896

**Published:** 2025-09-21

**Authors:** Nicholas Marks, Mya Abigail Acosta, Kristen Pickles, Scott McAlister, Katy J. L. Bell, the NSW Health Net Zero Clinical Leads Program

**Affiliations:** ^1^ NSW Health Net Zero Clinical Lead, Climate Risk and Net Zero Unit Ministry of Health St Leonards New South Wales Australia; ^2^ Calvary Mater Hospital Hunter New England Imaging, NSW Health Newcastle New South Wales Australia; ^3^ Wiser Healthcare Research Collaboration, School of Public Health, Faculty of Medicine and Health The University of Sydney Sydney New South Wales Australia; ^4^ Healthy Environments and Lives (HEAL) Global Research Centre, Faculty of Health, Health Research Institute University of Canberra Canberra Australian Capital Territory Australia; ^5^ The Healthcare Carbon Lab, Department of Critical Care University of Melbourne Parkville Victoria Australia

**Keywords:** carbon footprint, computerised tomography, diagnostic imaging, greenhouse gas emissions, net zero, power consumption, radiography

## Abstract

**Introduction:**

Medical imaging has been identified as a carbon hotspot in health care, and demand for imaging services is increasing. This study investigated switching off a surplus computerised tomography (CT) scanner when not clinically required as a possible simple and scalable intervention to reduce healthcare emissions.

**Methods:**

This before‐after quality improvement study introduced a ‘Switch it off’ intervention where radiography staff switched off the power to a surplus CT scanner after hours (17:00–08:00) for 7 days (‘intervention period’: 07/7/2023–13/07/2023). Using a power data logger, power consumption (kilowatt hours, KWh) during the intervention period was compared to 7 days without the switch‐off practice (‘control period’: 24/07/2023–31/07/2023). Financial and carbon emission impacts were calculated based on energy consumption. All CT radiographers working in the department were invited to undertake a pre and post intervention survey. Differences in quantitative data pre‐ and postintervention were analysed using chi‐squared test for independent proportions. Free text survey responses were summarised into themes.

**Results:**

Compared with energy use in the control period (433.96 kWh), there was a reduction in 139.79 kWh during the intervention period (294.17 kWh): 32% relative reduction. Extrapolation to 12 months found potential savings of 7280 kWh in energy use, $1381 to the hospital budget, and 5.5 T CO_2_e to the carbon budget. Of the 22 CT radiographers invited, 10 (45%) completed the survey, reporting no or trivial clinical impacts from switching off. The proportion of radiographers reporting switching off the scanner when not in use increased by 70% (95% CI: 39%, 100%; *p* = 0.002) from 10% (1/10) pre‐ to 80% (8/10) postintervention.

**Conclusion:**

Identifying and switching off surplus CT scanners in low use times is a simple and scalable intervention that can achieve significant power, financial and carbon savings with little to no impact on clinical workflow.

## Graphical abstract

A high proportion of the energy use of computed tomography (CT) scanners is while they are in an idle state. This quality improvement study investigated energy, carbon, financial and health service impacts of turning off a surplus CT scanner, when not clinically required, in a regional metropolitan hospital.

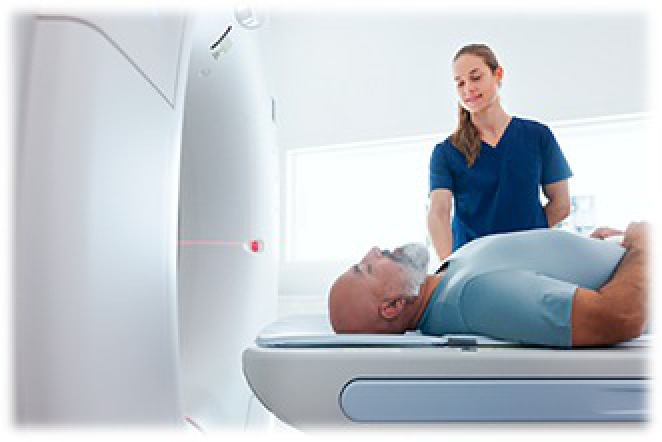

## 1 Introduction

There is growing concern around the impact that human activities have on climate change and planetary health, and recognition of the urgent need for mitigation and transition to net zero greenhouse gas emissions. Health care is no exception, currently contributing approximately 5% (2 gigatons) of global CO_2_e (carbon) emissions [1]—the equivalent of the 5th largest nation in the world. To avoid undermining its own mission of improving the population's health, health care must urgently decarbonise.

Medical imaging has been found to have a large carbon footprint and is estimated to contribute 1% of all global GHG emissions [2]. Imaging machines like magnetic resonance imaging (MRI), angiography suites and computed tomography (CT) scanners all use substantial amounts of electrical power at all hours. One department with 5 MRI scanners and 3 CT scanners uses the equivalent electrical power of 85 x four‐person households [3]. A high proportion of the energy use of CT scanners (66%) and MRI scanners (33%) is while they are in an idle state, between scans and overnight when not in use. The total energy used by a CT scanner in an idle state can be between 14 and 30 times higher than the energy used for active scans [3, 4]. A recent systematic review—with studies primarily from North America and Europe—reported that 40%–91% of energy consumed by radiological devices is when they are ‘on’ but not working. This review also identified turning off devices during idle periods and implementing workflow informatic tools as strategies to reduce energy use in radiology [5].

While emergency scanning necessitates constant availability of some medical imaging scanners, many scanners left switched on overnight are surplus to clinical requirements [6]. Varying levels of switching off are available on many CT scanners. Reduced power modes, where nonessential components are automated in a sleep mode, powering down the computer software and gantry controls, and switching off mains power entirely to the scanner and componentry are all options. In this study, powering down the computer software and gantry controls but leaving residual power to keep detectors at a base level heat for more efficient reboot/recalibration was the mode undertaken. Switching off underutilised scanners may be a simple measure to lower carbon emissions and financial costs without compromising patient health or clinical workflow [6]. With Australia ranking first globally in CT scanners per capita [7], switching off scanners presents a large opportunity for potential savings for the Australian health system.

This study aimed to assess, in a large tertiary metropolitan hospital in New South Wales, Australia, the impact of switching off a CT scanner outside normal scanning hours on power consumption, financial cost, carbon footprint, staff knowledge and attitudes and health service delivery.

## 2 Methods

The study was led by a NSW Health Net Zero Lead (principal investigator) with academic support from a University‐based team of public health researchers (co‐investigators) as part of the Wiser Healthcare Net Zero partnership [8].

### 2.1 Design

A before and after evaluation of a quality improvement study to ‘switch off’ one surplus CT scanner when not in use. Mixed methods analysis was used to summarise the quantitative data (power, financial, carbon emissions, quantitative survey data) and qualitative data (free text survey responses).

### 2.2 Setting and Procedure

An audit of all hospitals in the local health district was undertaken to determine opportunities for powering down CT scanners during low use times. Data collected included: CT scanning operational hours, types of scanners, staff coverage and any routine periods of inactivity. A large tertiary public hospital was identified as the most suitable study site for two main reasons: (1) the hospital had three CT scanners, so maintaining a full clinical service for emergency scanning was feasible while switching off one underutilised scanner outside of normal business hours (08:00–17:00), and (2) engineering staff were available on‐site to assist with the placement of the power logger (and the hospital had multiple loggers available for use).

A nine‐question preintervention survey was drafted by the lead investigator and further refined by the study team (Appendix S1). The purpose of this survey was to explore the radiographers' knowledge and attitudes about CT energy expenditure, CT scanner usage and switch‐off behaviour, suitable scanners/opportunity for intervention and barriers and willingness to switch off CT scanners overnight. An email was sent to all CT radiographers working in the department with a link to the survey, and a poster with a QR code linking to the survey was also placed in the CT work area. Through the preintervention survey and consultation with senior radiographers and imaging management staff at the hospital, one CT scanner was identified that was predominantly used only during daytime office hours as the target for our intervention. This scanner was a Canon Aquilion Prime SP that had been in service since 2016. Its primary purpose was servicing requests for scans from the hospital's outpatient clinics. It had a routine booked clinical worklist during the hours 08:00–17:00 Monday to Friday. It also served as an overflow scanner for emergency and ward inpatients as required. Preintervention it was left on 24 h a day, 7 days a week and was reset with a daily reboot at the beginning of each clinical day at approximately 08:00. This was to clear log files, and it was then run through a normal warm‐up and daily calibration procedure. This CT scanner takes approximately 5 min to switch off and approximately 8 min from switch on; after this time a tube warm‐up of a further 8 min is encouraged by the manufacturer and partially automated during switch on. Thus, the time from switching on until ‘ready to scan’ was 16 min. The local Canon engineer was consulted prior to this study to confirm the scanner's suitability given that switching off some types of CT scanners for prolonged periods can have implications for compliance with manufacturer recommendations for optimal scan conditions.

### 2.3 Intervention

All CT radiographers (*n* = 22) in the department were emailed summaries of data from previous studies outlining the impact of CT power consumption and the carbon footprint of imaging. Posters (Appendix S2) were also emailed to all CT radiographers and placed within the CT console room as a visual reminder to switch off the scanner at the end of the clinical day. Staff were also informed that they were permitted to switch on the scanner if clinically required.

During the 7‐day control period (‘scanner on’) there was no change to daily workload or restart and shutdown procedures. During the 7‐day intervention period (‘scanner off’), the radiographers were asked to switch off the scanner at the end of the clinical worklist each day and switch the scanner on again the next morning. Clinical workload was not altered at any time in the study period, and required scans were booked by staff as needed. The exact switch off and on times each day varied according to clinical workload and staff availability.

A Fluke 135 data logger (an electronic device) was installed inside the switchboard that supplies power to the CT scanner by trained staff (electricians/engineers) during the normal daily shutdown. The logger was installed on July 1st 2023, and the intervention period (‘scanner off’) data were acquired from July 7th–14th 2023. Due to technological issues with the power logger during the initial preintervention data collection, the control (‘scanner on’) data were acquired postintervention from July 24th–31st 2023.

The power logger read the amount of electrical current, voltage and overall power supply for a set period of time at specific time intervals. CT power consumption was recorded every 2 min and 20 s for both intervention and control periods. The scanner power use was categorised into different energy states: active scanning, idle state and system off. Active scanning indicates the scanner is acquiring data and in the process of planning and preparation, idle state indicates the scanner is switched on and between active scans, and system off indicates the scanner is switched off and unable to scan. A timeline illustrating the data collection process is included in Appendix S3.

After the intervention, an eight‐question postintervention survey was emailed to all CT radiographers in the department. This survey contained four questions that were asked in the pre‐intervention survey, with the aim to capture changes to practice, number of staff switching off scanners and staff knowledge or attitudes about ‘switching off’. The purpose of the remaining questions in the postintervention survey was to capture any unintended effects of the intervention on staff or patient care (Appendix S1). A question regarding what stopped staff switching off the scanner was removed from the postsurvey as the intervention was to switch off the scanner and thus this question became redundant.

Power consumption data (measured in KWh) collected via power logging the CT scanner was used to calculate financial and carbon savings. Power was supplied to the hospital by local power suppliers. Financial costs ($AUD) were calculated using a conversion factor of $0.19/kWh as provided by the hospital's building services (this accounts for financial savings resulting from the installation of a solar system in late 2021). Environmental impacts were calculated using the National Greenhouse Accounts Factor in kg Carbon Dioxide equivalent (CO_2_e) for electricity in NSW use of 0.68/kWh. At the time of the study, approximately 80% of the NSW electricity grid was from coal. This is calculated into the NGA factors.

### 2.4 Analysis

A ‘triple bottom line’ adapted for health care was used to assess impacts of the intervention across health, financial and environmental domains [9]. Standard descriptive statistics (means, medians, maximum and minimum) were used to summarise power consumption data. Graphical techniques were used to visualise data and to observe differences. Staff attitudes and health service delivery impacts measured via the pre‐ and postintervention survey described above were analysed using chi‐squared test for independent proportions, and free text responses were summarised into themes. Statistical analyses were conducted in SAS Studio 3.81 (SAS 9.4). Excel was used for graphical display.

### 2.5 Ethical Approval

This quality improvement project, including radiographer survey, was granted exemption from the Hunter New England Local Health District Human Research Ethics Committee review (Authorisation number: AU202212‐05). No patients were recruited in this study, and there were no participant information statements or consent forms. Return of the surveys from the staff was considered consent to participate.

## 3 Results

During the 7‐day control period, there were 64 CT scans, and during the 7‐day intervention period, there were 49 CT scans. The difference in workload was attributed to bookings, staffing levels and clinical requirements. No scans were conducted on the weekend (Friday afternoon to Monday morning) during either period, nor after hours, but the machine was only switched off during the intervention weekend (from Friday 16:58 until Monday 08:00) and the intervention weekdays after hours (see Table 2 for specific timings).

In the control period, the scanner was rebooted daily as part of the normal daily procedures designed to maintain optimal functioning and clean up software and storage logs as per manufacturer recommendations. Apart from this brief period during reboot, the scanner remained on throughout the 7‐day data collection. The time taken to reboot the scanner in the study was approximately 8 min. When combined with the calibration time, this accounts for 16 min per day.

During the intervention period, the switch off and on time varied every day according to clinical load, the staff working that day, and the availability of staff to manually switch off the scanner (Table 2). The scanner was switched off overnight each weekday and remained off until turned on to start the next day. It was also switched off for the full 24 h each day of the weekend. Aside from the weekend, the shortest time period for the machine being on was 8 h and 40 min, and the longest period was 14 h and 8 min. The median time the scanner was on across the 7‐day intervention period was 9 h 37 min, and the mean time on was 7 h and 42 min.

### 3.1 Power Consumption

**FIGURE 1:**
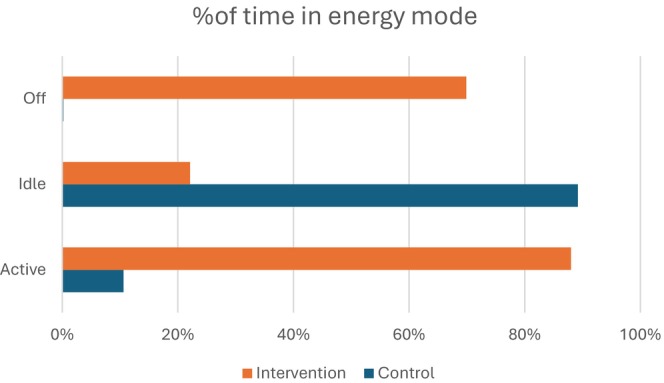
Proportion of time the scanner spent in active, idle and off states in intervention and control periods.

**FIGURE 2:**
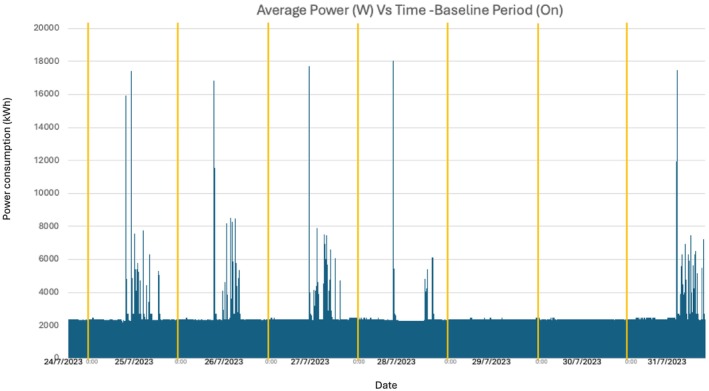
Energy use during control period.

**FIGURE 3:**
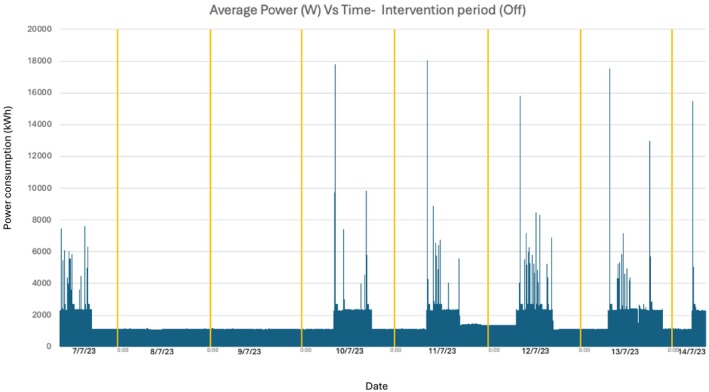
Energy use during intervention period.

The percentage of time spent in each scanner state is shown in Figure 1. During the control period, 10.6% of the time was in active scanning, 89% in idle state and 0.4% in system off (during daily reboot). During the intervention period, 8.8% of the time was in active scanning, 21.2% in idle state and 70% in system off. The power consumption in control and intervention periods is shown in Figures 2 and 3, respectively. In active scanning, power consumption varied according to the length and type of scan being undertaken. In idle state, power consumption was steady at approximately 2.3 kWh. In system off during the intervention period, power consumption was steady at approximately 1.1 kWh (this power consumption is for keeping the detectors warm so that when the system is switched back on the detectors are capable of resuming scanning).

Daily power usage and total scans during the control period are shown in Table 1, and power readings are shown in Figure 2. During the control period, the total power consumption was 433.95 kWh over 7 days. The mean and median power consumption per day were 62 kWh and 63.17 kWh, respectively. Differences in the number of scans completed per day did not appear to have a large impact on the power used. The lowest power consumption was over the weekend when the scanner was left on but unused, with a mean consumption of 58 kWh per day. Total power usage for the control weekend was 156.33 kWh.

**TABLE 1:** Summary of energy used by the CT scanner during control and intervention periods.

Date	Total power	Time logged	Scans done
Control period			
Day 1: Monday	18.63 kWh	7 h 40 min	0
Day 2: Tuesday	64.79 kWh	24 h	12
Day 3: Wednesday	63.17 kWh	24 h	12
Day 4: Thursday	63.64 kWh	24 h	19
Day 5: Friday	60.3 kWh	24 h	5
Day 6: Saturday	58.5 kWh	24 h	0
Day 7: Sunday	58.59 kWh	24 h	0
Day 8: Monday	46.33 kWh	16 h 20 min	16
Total for week	433.95 kWh	168 h	64
Intervention period			
Day 1: Friday	32.0 kWh	15 h 10 min	13
Day 2: Saturday	29.2 kWh	24 h	0
Day 3: Sunday	29.2 kWh	24 h	0
Day 4: Monday	43.5 kWh	24 h	4
Day 5: Tuesday	45.4 kWh	24 h	7
Day 6: Wednesday	48.5 kWh	24 h	16
Day 7: Thursday	50.2 kWh	24 h	7
Day 8: Friday	16.2 kWh	8 h 50 min	2
Total for week	294.2 kWh	168 h	49

*Note:* Due to data logger availability and staff availability to instal, the logger the control period began at 1620 on Monday 24/07/2023. To complete the 7‐day recording of data, the logger was disconnected at 1620 on Monday 31/07/2023.

Daily power usage and total scans during the Intervention period are shown in Tables 1 and 2, and power readings are shown in Figure 3. During the intervention period, the total power consumption was 294.2 kWh over 7 days. The mean and median power consumption per day were 42 kWh and 45.3 kWh, respectively. The highest kWh occurred on the day when the scanner was left in ‘on’ mode for the longest period of time (14 h and 8 min), with a total daily consumption of 50.19 kWh. The lowest kWh was during the weekend, with a mean daily consumption of 29 kWh. The total power usage for the intervention weekend, where the scanner was switched off, was 76.66 kWh: 80 kWh lower than the control weekend when the scanner was not switched off.

**TABLE 2:** Energy used by the CT scanner when switched on and off during the intervention period.

DATE	Measurement interval (via logger)	Time on	Time off	Elapsed time scanner on	Scans done	kWh for 24 h	kWh when on	kWh when off
Day 1: Friday 7	08:50 to 24:00	08:50	16:58	8 h and 8 min	13	32	23.5	8.6
Day 2: Saturday	0:00 to 24:00	Off	Off	0 h	0	29.2	0	29.2
Day 3: Sunday	0:00 to 24:00	Off	Off	0 h	0	29.2	0	29.2
Day 4: Monday	0:00 to 24:00	08:00	17:48	9 h and 48 min	4	43.5	26.1	17.3
Day 5: Tuesday	0:00 to 24:00	08:00	16:40	8 h and 40 min	7	45.4	24.6	20.8
Day 6: Wednesday	0:00 to 24:00	07:24	17:01	9 h and 37 min	16	48.5	28.9	19.5
Day 7: Thursday	0:00 to 24:00	07:22	21:30	14 h and 8 min	7	50.2	38.1	12.1
Day 8: Friday	0:00 to 08:50	05:11	08:50	3 h and 39 min	2	16.2	9.8	6.4
Total				54 h for the week	49	294.2	151	143.1
			Mean time scanner switched on for the week 7 h 42 min per day					

*Note:* The number of scans had minimal impact on energy use compared to the time in which the scanner is switched off. The busiest clinical day (Wednesday) was not the day with the highest energy use. This occurred on the Thursday where the scanner was left on (and inactive) for an extra 5 h. Although active scanning uses large amounts of power, the scan times are extremely short. The constant draw of power over prolonged periods with scanner idle results in greater cumulative power use. Due to data logger and staff availability to instal the machine the logger for the intervention started measuring at 0850 on Friday 07/07/2023, resulting in only 15 h 10 min of data, whereas each of Monday 10/07/2023 through to Thursday 13/07/2023 had 24 h of data. This resulted in an apparently lower power use on Friday 07/07/2023 that was artefactual (measurement interval was shorter by approximately one third). To complete the week the logger was disconnected Friday 14/09/2023 at 0850, resulting in only 8 h 50 min of measurement for this day.

For both the control and intervention periods, there were large power spikes at the beginning of each day due to the daily tube warm‐up. The tube warm‐up is run to optimise scanning conditions as per manufacturer instructions and is a process that runs through a set number of imaging exposures prior to clinical scanning.

### 3.2 Financial Cost

Compared with the control period ($82.45), costs were reduced during the intervention period ($55.89), resulting in $26.56 saved for 1 week. This represents a 32% relative reduction in costs. Extrapolating this to savings from switching off the single CT scanner when not in use for a period of 1 year results in potential savings of $1381 AUD.

### 3.3 Carbon Emissions

Compared with carbon emissions during the control period (295 kgCO_2_e) carbon costs were reduced during the intervention period (200 kgCO_2_e), resulting in 95 kgCO_2_e savings for 1 week. This represents a 32% relative reduction in carbon costs. Almost half of the carbon savings occurred during the weekend period, with carbon savings of 40 kgCO_2_e. Extrapolating this to savings from switching off the single CT scanner when not in use for a period of 1 year would result in 4.95 t in CO_2_e carbon savings. This equates to the average carbon footprint of two light vehicles operating for a normal 1‐year period [10].

### 3.4 Staff Knowledge, Attitudes and Health Service Delivery Impacts

10/22 (45%) CT radiographers who were invited completed the survey. Quantitative responses and analysis of the survey results are shown in Table 3 and Appendix S4. Preintervention, 10% of respondents stated that they switched off scanners when not in use for an extended period. Explanations provided for why scanners were not switched off more often varied between never have done it to not thinking there was an opportunity (Appendix S5). Respondents also stated that they were unaware of what the power consumption of CT was, with only 10% preintervention and 30% postintervention identifying that most CT energy consumption is in idle mode between patients and overnight in low use periods.

**TABLE 3:** Quantitative survey responses preintervention vs. postintervention.

Question	Response option	Preintervention	Postintervention	Difference in proportions^b^
When do you currently switch off any of your CT scanners?	When not being used for an extended period^a^	1 (10%)	8 (80%)	70% (95% CI: 39%, 100%) *X* ^2^_1_ = 9.90, *p* = 0.002
	Daily reboot only	9 (90%)	2 (20%)	
Are there any potential opportunities you can think of, to switch off any of your CT scanners?	Yes^a^	10 (100%)	8 (80%)	−20% (95% CI: −45%, 5%) *X* ^2^_1_ = 2.22, *p* = 0.136
	No	0 (0%)	2 (20%)	
Did you know most CT energy consumption is in idle mode between patients and overnight in low use periods?	Yes^a^	1 (10%)	3 (30%)	20% (95% CI: −14%, 54%) *X* ^2^_1_ = 1.25, *p* = 0.264
	No	3 (30%)	7 (70%)	

*Note:* *N* = 10.

^a^ Denotes response proportion compared preintervention and postintervention.

^b^ % rounded to 0 dp.

The proportion of respondents who stated that they switched off the intervention scanner when it was expected to be unused for an extended period increased by 70% (95% CI: 39%, 100%; *p* = 0.002) from 10% pre‐ to 80% postintervention. All respondents attributed this change in behaviour to the educational resources provided with the intervention. Staff identified potential opportunities to switch off CT scanners both preintervention (100%) and postintervention (80%), particularly overnight or when workflow permits. Concerns about switching off scanners included the potential for failures on start‐up or needing the (off) scanner urgently for emergency patients. Two respondents identified instances where staff could have used the scanner that was switched off during the project, for example, the 1 day when the patient list was busy.

All 10 respondents indicated they had some concerns or were very concerned about the carbon footprint of imaging and CT scanners. Comments highlighted personal awareness about resources being wasted, positive attitudes towards reducing unnecessary power consumption by switching off equipment and motivation to make as little environmental impact as possible.

## 4 Discussion

This is the first Australian study to investigate the beneficial impacts from switching off radiology equipment when not in use. It showed that the simple measure of switching off CT scanners that are not clinically required can lead to considerable power savings with associated carbon and financial savings. Over the course of a 7‐day intervention period, a 32% reduction in daily power consumption was observed from switching off a single CT scanner when not in use. Over the weekend period where no scans were done, there was close to a 50% reduction in power use. Switching off the CT scanner when not in use had a much greater impact on power consumption than the number of CT scans done per day. This resulted in reduced financial and carbon costs, with minimal impact on clinical workflow. Extrapolation to 12 months found potential savings of 7280 kWh in energy use, $1381 to the hospital budget, and 5.5 T CO_2_e to the carbon budget.

A systematic search of the literature was undertaken to identify published reports on interventions to reduce the energy consumption of imaging or ancillary equipment on power consumption, financial costs and greenhouse gas emissions [11]. This identified 7 studies, all published between 2021 and 2023, and none from Australia (Canada *n* = 2, Switzerland *n* = 2, Germany *n* = 1, France *n* = 1, Denmark *n* = 1). In keeping with the findings of the current study where the CT was in ‘idle’ state 88% of the time, a US study of a scanner service offering similar routine hours found that the CT room was unoccupied 72% of the time [4]. The US study found that only 4% of used energy was during active scan times, similar to this study's finding of 6%–7% of energy use.

A Canadian study reporting on the effects of switching off a dual‐source CT scanner servicing outpatient clinics after normal business hours also found carbon and cost savings [6]. They also observed some savings overnight and greater savings across a weekend period when no scanning was required. Because of the higher energy use for the model of scanner in the Canadian study, the power savings observed were greater than those reported in this study. Interestingly, due to the difference in energy source between this Australian study (largely coal based energy) and the Canadian study (largely renewable energy), a greater carbon savings were observed in the current study despite saving less power. This highlights the importance of facility/institutional choice of Scope 2 energy provider, where choice is available.

Quality improvement studies in Germany and Switzerland have also found energy, carbon and financial savings from switching off equipment. Buttner et al. implemented a switch off plus reminders intervention for computers and monitors [12] and Heye et al. developed a workflow to ensure the powering down of devices (included 60 medical imaging systems, PACS, computers, monitors, printers) [3]. These studies estimated annual energy savings in their departments of 72,337 kWh [3] and 109,021 kWh [12] following intervention, carbon savings of 9.26 T CO_2_e [3] and 3.2 T CO_2_e [12] per year, and annual cost savings of $19,531–$60,937 [3] and $USD 2100 [12]. Two studies calculated hypothetical energy savings based on operational adjustments or optimised scenarios in imaging departments. For example, Barloese et al. calculated hypothetical savings based on recorded usual activity of a CT, MRI and PET/CT scanner in Denmark [13], while Vosshenrich et al. evaluated the impact of two hypothetical operational scenarios where equipment was in an off state for 12 h overnight all days of the week versus for 12 h overnight on weekdays and 48 h on weekends [14].

Participants in the Australian study were unanimous in their concern about the environmental impact of medical imaging. This matches findings from the recent 2023 NSW Health People Matter Employee survey where 80% of NSW Health staff were interested in sustainable health care [15].

### 4.1 Strengths and Limitations

A key strength of this study is its applicability to other Australian clinical radiology settings, particularly to major tertiary hospitals with 24‐h imaging requirements. Staff involvement was integrated into normal clinical roles and scanning capacity, and the intervention had limited clinical and functional impacts. In the post intervention survey staff identified further opportunities to switch off underutilised scanners and equipment, increasing potential savings and project impact. There was continued enthusiasm for staff involvement in optimising scanner use, highlighting the potential for commitment and long‐term continuation of the ‘switch it off’ practice. The study did not measure other power consumption related to the intervention, including adjacent cooling requirements of the scanning machines, room climate control and lighting and computer usage. Comparing their power consumption during the intervention and the control period would likely indicate greater saving than those identified in the study.

This study had several limitations. Potential confounders, including the variability in number of scans and clinical workload, were not adjusted for in the analysis. Due to practical constraints, power consumption logging was limited to only two 7‐day periods. Whether clinical workflow would remain unimpacted across a longer period of ‘switch it off’ is uncertain. However, staff feedback and results from the postintervention survey suggest minimal impact on clinical duties and an interest in continuing the practice. The interval between power logging measurements was also longer than the similar study in Canada [6]. This may have led to small changes in energy states not being as precisely captured in the active scanning times. However, this is likely to have had minimal impact on the estimated costs and carbon savings which largely occurred during uniform scanner energy states—on or off.

To lessen the impact to clinical staff and to achieve a real‐world analysis, fixed times for switching the scanner on and off during the intervention were not established. Instead, staff were left to switch off the scanner according to clinical demand and at their own discretion. Despite the short time needed to switch the scanner off, the handover between shift changes and the requirement of other clinical scans to take precedence over the study, the times at which the scanner was switched on and off varied considerably across the data collection period. As staff become more accustomed to the process and build it into handover and daily workflow, a more uniform time frame may be achieved, resulting in less daily variation. Data collection was limited to one scanner vendor and model. There is a wide range of power consumption across different scanner types due to differences in clinical options and hardware. Lower energy modes and automation capabilities also differ, potentially affecting estimates of power consumption and subsequent savings from what we observed in this study. The scanner was not needed to be turned on overnight in our study. If the scanner was turned on and off several times overnight, then warm‐ups required would increase the power usage, reducing power savings.

#### 4.1.1 Implications for Policy and Practice

The make and model of CT scanners significantly impact power consumption. While the scanner chosen for this study was selected to minimise clinical impact, opportunities may exist to switch off higher consumption scanners. Manufacturer data may help identify scanners with higher power consumption. Some scanners may achieve the savings observed in this study in a fraction of the time switched off, as power consumption can vary by approximately six times according to vendors' data (personal correspondence). Sites with multiple scanners should aim to maximise use of one scanner to allow others to be switched off. Staff may also identify other equipment that could be switched off. Reduced use of scanner cooling, HVAC and heat control, computers and lighting can all contribute to lessen the energy consumption of entire imaging departments. Staff can also review scanner settings to optimise efficiency and engage automation for lower power modes where feasible. The scanner used in this study keeps heat to the detector at a base level even when the machine is switched off at the PC and gantry. This is why the scanner is still utilising power even when in an ‘off‐state’. While vendors are actively improving low power modes, switching off scanners will always lead to greater power savings. Furthermore, vendor data can inform future equipment purchases for maximum efficiency, aligning with sustainability goals in purchasing decisions. The purchase of further scanners should be assessed in a sustainability standpoint in conjunction with the clinical demand [16].

Departmental heads can conduct internal audits to assess equipment left on after hours and the frequency that equipment is used. This may identify opportunities for power savings and financial and carbon emission savings. Engaging clinical staff in sustainability quality improvement projects such as this may sustain changes in practice and long‐term impact. As technology improves, the ability to establish real‐time displays or dashboards of the consumption of the entire department may be possible. In this way, staff may be able to see their impact in real time, providing positive feedback to sustain practice change.

The savings in energy consumption, financial costs and carbon footprint for switching off one CT scanner give an indication of the potential savings if this simple intervention was rolled out to other sites. The high number of scanners in Australia suggests there may be significant opportunities for collective savings with the uptake of the switch it off intervention at scale. For example, within this Local Health District alone, 15 scanners are in service, including the one used in the study. In the time since the study was completed, two further scanners at other hospitals in the LHD have been identified as suitable for the intervention in addition to the one in this study. This means that now 3 of the 15 scanners (20%) in the LHD are now switched off overnight. If similar proportions of scanners were suitable for the intervention across New South Wales, then 23 of the 115 plus scanners in use in hospitals across the state could be switched off at night and weekends—resulting in carbon and financial savings many times greater than that reported for a single scanner. After more than a year since implementing the intervention, no problems have been reported at any of the sites from regularly turning off the surplus scanners when not in use.

Not all sites and scanners will have the capacity to operate within this system. CT is an on‐demand emergency service where timely scans and results are essential, particularly in trauma and stroke imaging. In sites using an emergency on‐call system, there are time constraints when scanners need to be switched on from off, impacting both patient welfare and staff who must travel to the site, often at inhospitable overnight times. This issue can be further investigated and potentially addressed by involving on‐site staff in switching on scanners, encouraging vendors to improve activation times, or implementing remotely accessible power systems. Regardless, scanners should be investigated to ensure that low power modes are activated wherever possible so that the least power consumption is occurring whenever possible. Further research may also explore facilitators and barriers to sustaining this practice change as well as possible expansion into switching of scanners of other modalities and ancillary equipment.

## 5 Conclusion

This quality improvement study found the potential for significant carbon and energy savings from a simple intervention of switching off a CT scanner when not in use. Educating and empowering clinical staff to think about possible downtimes and workloads means that they engage with the initiative and are willing to find and utilise switch‐off times for the CT system best suited to their clinical environment. This leads to real and measurable savings in both carbon footprint and financial costs while limiting the downtime to staff and clinical scanning.

Collective ownership of the project by the frontline clinicians means the changes have been sustained after completion of the study, and radiographers at other local sites have also now started identifying and switching off surplus scanners. While the savings at individual sites are modest, when extrapolated across the entire imaging community, collectively these small changes can make a significant impact towards achieving Net Zero health systems.

## Ethics Statement

The study granted exemption from Hunter New England Local Health District Human Research Ethics Committee review (Authorisation number: AU202212‐05).

## Conflicts of Interest

The authors declare no conflicts of interest.

## Supporting information


Appendices S1–S5

## Data Availability

All data are available in the manuscript and Supporting Information.
