# Supplementary material for: Switch It Off! Carbon, Financial and Health Service Impacts of Switching Off a Computed Tomography Scanner: A Quality Improvement Study
Source: J Med Radiat Sci. 2025 Sep 21;72(4):430–8. doi: 10.1002/jmrs.896 (PMC12661081; doi:10.1002/jmrs.896)
Supplement: Supplementary file 1 — Appendices S1–S5 [file JMRS-72-430-s001.docx]

# Supporting information

**Appendix S1: Survey Questions**

| Pre-intervention survey | Post-intervention survey |
| --- | --- |
|  | How many CT scanners do you operate?   - 1 - 2 - 3 |
| Do you currently switch off any of your CT scanners?   - Never - For daily reboot only - When not being used for an extended period - Other (please specify) | Do you currently switch off any of your CT scanners?   - Never - For daily reboot only - When not being used for an extended period - Other (please specify) |
| What stops you switching off scanners overnight?   - Not routine/never have - No need to/no benefit - Fear of consequences - Don’t know how - No opportunity to/too many scans - Been advised not to - Other (please specify) |  |
| Do you think there is any potential opportunity to switch off any scanners given your current workflow/clinical practice?   - Yes – Canon only (1900-0800) - No - Canon scanner plus 1 of the Siemens scanners (what hours?) | Are there any potential opportunities you can think of, to switch off any of your CT scanners?   - Yes - No |
| Would you start switching off scanners if instructed and the opportunity is available?   - Yes - No |  |
| What concerns do you have if you switched off one or two of your CT scanners overnight?   - Patient wait time increases - Staff unhappy with unavailability - Fear scanner wont restart properly - Unsure how to switch on/off - Concern with switch on/warm up time - No concern/would switch off |  |
| Do you know how much energy expenditure is involved in CT and medical imaging as a department/modality?   - Yes - No |  |
| Did you know most CT energy consumption is in idle mode between patients and overnight in low use periods?   - Yes - No | Did you know most CT energy consumption is in idle mode between patients and overnight in low use periods?   - Yes - No |
| Are you concerned about energy consumption of CT/medical imaging as a whole?   - Yes - No | Are you concerned about energy consumption of CT/medical imaging, and the carbon footprint of this?   - No concerns - Some concerns - Very concerned |
| Do you know anything about HNE and NSW Health commitment to move to a NetZero health service?   - Yes - No |  |
|  | Were there any unintended clinical impacts due to the project? |
|  | Was there push back from staff external to imaging and what was it? |
|  | Do you have any other comments about the study or suggestions for further work in this area? |

**Appendix S2: Poster for Switch it Off Intervention**


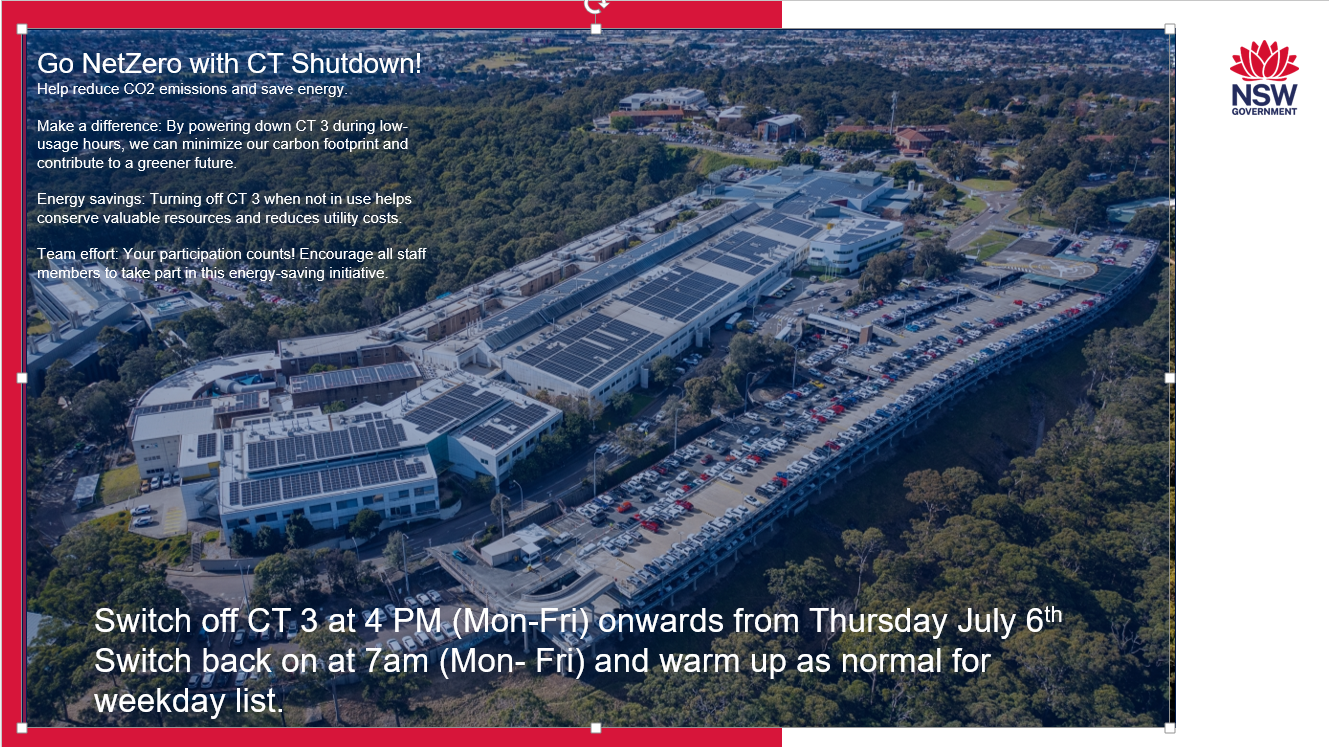


**Appendix S3: Study timeline**

**
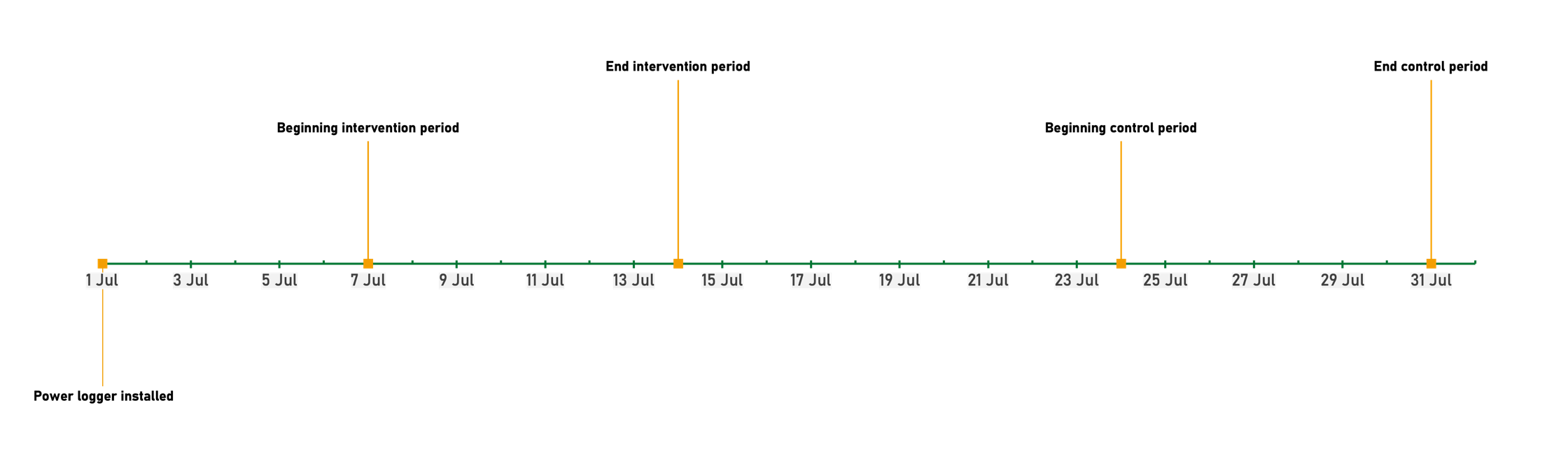
**

**Appendix S4: Survey responses**

**
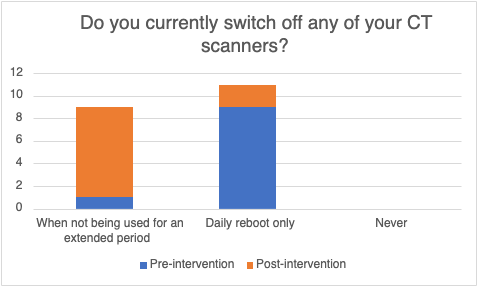
**

**
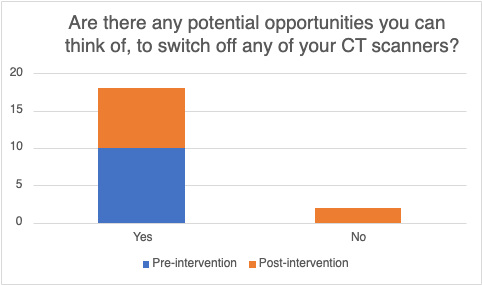
**

**
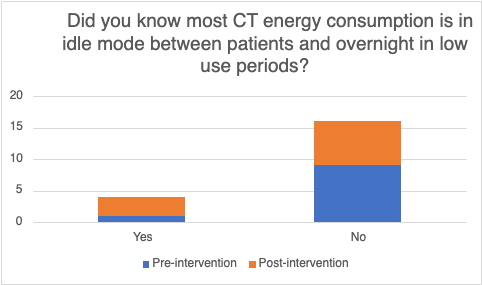
**

**Appendix S5: Free text survey question responses:**

| Please tell us about what prompted you to switch off your scanner | Net Zero trial and when at risk during storms  Scanner 3 turn off trial  Nick’s study  Switch off the scanner 3 outpatient overnight  The trial undertaken and the attached info. No consequences thus far  Power saving benefits  Switch CT3 off overnight and if workflow permits, CT2 on nightshift only  For environmental reasons we have been turning CT3 (outpatient scanner) off from approx. 4pm-8am overnight |
| --- | --- |
| Please tell us more about the potential opportunities to switch off your scanner(s) | CT 3 Routinely Possibly CT2 if workload and workflow optimised  Power savings  CT 2 could possible be switched off once evening shift numbers are under control  One day of three  Perhaps opportunities to make overnight scans on one scanner only? Sometimes need 2 though.  CT 3 could be switched off overnight  Switch CT 3 off |
| Do you have any concerns about switching off any of these scanners? | Failures can occur on startup in our experience when full diagnostics are running  Not for the CT 3 scanner  Needing the scanner urgently for emergency patients  CT 1 & 2 should remain on other than daily reboot. CT3 should be on until 8pm perhaps  No |
| Were there any unintended clinical impacts due to the project? | One day the list on CT1/CT2 was busy and we were going to get a CT trained rad from generals to do some scans on CT3 to reduce the list but it was already turned off. Would be good for rad turning machine off to check it isn't needed still by afternoon staff  Occasionally there was a need for CT3 after 4pm if busy and a CT person in general able to help... but only until 8pm  No |
| Please tell us more about why you answered ‘some concerns’ (about energy consumption of imaging) | Personal awareness of costs and resources wasted  3 scanners running 24/7 is unnecessary  Could probably be reduced  Every little bit helps  It uses a lot of energy. there are chances to reduce this  We could switch off equipment and lights when not in use  Just like to conscious decision to make as little impact as possible  Definitely a positive for CT3 to be turned off when not in use/required to reduce its negative impact on the environment |
| Do you have any other comments about the study or suggestions for further work in this area? | Look at low hanging fruit. eg saving in energy on Lights, A/C and PCs on in use overnight, or low use rooms eg storerooms  Great idea – happy to expand if possible |
